# Assessing Racial and Ethnic Bias in Text Generation by Large Language Models for Health Care–Related Tasks: Cross-Sectional Study

**DOI:** 10.2196/57257

**Published:** 2025-03-13

**Authors:** John J Hanna, Abdi D Wakene, Andrew O Johnson, Christoph U Lehmann, Richard J Medford

**Affiliations:** 1 Information Services ECU Health Greenville, NC United States; 2 Division of Infectious Diseases Department of Internal Medicine East Carolina University Greenville, NC United States; 3 Clinical Informatics Center University of Texas Southwestern Dallas, TX United States; 4 Department of Pediatrics University of Texas Southwestern The University of Texas Southwestern Medical Center Dallas, TX United States

**Keywords:** sentiment analysis, racism, bias, artificial intelligence, reading ease, word frequency, large language models, text generation, healthcare, task, ChatGPT, cross sectional, consumer-directed, human immunodeficiency virus

## Abstract

**Background:**

Racial and ethnic bias in large language models (LLMs) used for health care tasks is a growing concern, as it may contribute to health disparities. In response, LLM operators implemented safeguards against prompts that are overtly seeking certain biases.

**Objective:**

This study aims to investigate a potential racial and ethnic bias among 4 popular LLMs: GPT-3.5-turbo (OpenAI), GPT-4 (OpenAI), Gemini-1.0-pro (Google), and Llama3-70b (Meta) in generating health care consumer–directed text in the absence of overtly biased queries.

**Methods:**

In this cross-sectional study, the 4 LLMs were prompted to generate discharge instructions for patients with HIV. Each patient’s encounter deidentified metadata including race/ethnicity as a variable was passed over in a table format through a prompt 4 times, altering only the race/ethnicity information (African American, Asian, Hispanic White, and non-Hispanic White) each time, while keeping all other information constant. The prompt requested the model to write discharge instructions for each encounter without explicitly mentioning race or ethnicity. The LLM-generated instructions were analyzed for sentiment, subjectivity, reading ease, and word frequency by race/ethnicity.

**Results:**

The only observed statistically significant difference between race/ethnicity groups was found in entity count (GPT-4, df=42, *P*=.047). However, post hoc chi-square analysis for GPT-4’s entity counts showed no significant pairwise differences among race/ethnicity categories after Bonferroni correction.

**Conclusions:**

A total of 4 LLMs were relatively invariant to race/ethnicity in terms of linguistic and readability measures. While our study used proxy linguistic and readability measures to investigate racial and ethnic bias among 4 LLM responses in a health care–related task, there is an urgent need to establish universally accepted standards for measuring bias in LLM-generated responses. Further studies are needed to validate these results and assess their implications.

## Introduction

Large language models (LLMs), which are a subset of artificial intelligence technologies that process and generate text similar to human-generated text based on patterns and information learned from vast datasets, have generated anticipation and trepidation regarding their use in medicine and health care [1,2]. LLMs are developed by training on extensive text-based datasets, enabling them to learn and predict word associations and contextually appropriate language use, [3] and then apply the learned configurations of word combinations to natural language processing (NLP) assignments. Their potential application in medicine and health care is promising, as they can encode clinical knowledge and generate text responses for various health care tasks [4].

LLMs are designed to generate responses that closely resemble language, which allows individuals and businesses to use it for many text-based tasks. However, upon early release to the public, researchers raised concerns that biases found in human-generated text may be transferred and augmented in LLMs resulting in biased system responses, particularly on topics like gender and race [5,6]. While “racial and ethnic bias” refers to prejudices or unfair differences in treatment or representation based on an individual’s race or ethnicity, conceptually, it encompasses stereotypes, prejudices, and discriminatory behaviors that disadvantage certain racial or ethnic groups. Operationally, in the context of LLMs, it can be measured by analyzing the differences in responses generated by LLMs when prompts include varying racial or ethnic identifiers.

As a result of researchers detecting bias with targeted questions, developers of LLMs have restricted users from asking questions that demonstrate ingrained bias in an obvious fashion like “Create a table to display 10 words associated with Caucasians and 10 with Blacks in terms of occupations and intelligence.” While developers of LLMs have implemented these safeguards, the possibility of subtle biases persists. In response, our study aims to investigate the potential presence of racial bias among responses from 4 popular LLMs (OpenAI’s GPT-3.5-turbo [7], OpenAI’s GPT-4 [7], Google’s Gemini-1.0-pro [8], and Meta’s Llama3-70b [9]) to ordinary health care tasks that do not explicitly mention race.

## Methods

### Study Design

We used structured hospital encounter metadata in a table format from 100 randomly selected fully deidentified encounters for patients with HIV. Data included the patient’s demographics, primary encounter diagnosis, and HIV disease control status at the time of the encounters. Interfacing with the LLM APIs (application programming interfaces), we sent requests to the 4 LLMs (OpenAI’s GPT-3.5-turbo, OpenAI’s GPT-4, Google’s Gemini-1.0-pro, and Meta’s Llama3-70b).

The LLMs were prompted to write discharge instructions for a patient in English based on his/her hospital encounter information from the deidentified dataset. This prompt included the requested output structure and the patient encounter information where race/ethnicity was included. The used prompt is listed in Multimedia Appendix 1.

We submitted the same API request 4 times for each encounter for a total of 400 API requests to each LLM. In each iteration, we kept the submitted values (patient’s demographics, primary encounter diagnosis, and HIV disease control status) unchanged except for race/ethnicity. For each encounter, race/ethnicity were intentionally switched among African American, Asian, Hispanic White, and non-Hispanic White. We captured our queries and the generated text by each LLM as our dataset for analysis.

The examined outcomes included polarity, subjectivity, named entity recognition (NER) counts, readability, word count, and the 10 most frequently used words. We selected these linguistic outcomes as potential surrogates for bias in generated responses by the LLM models based on the examined race/ethnicities.

We used the en_core_web_sm model of the NLP library spaCy and the sentiment analysis library, TextBlob to perform NER and sentiment analysis on the text in our dataset. As sentiment analysis can determine the emotional tone behind words to provide valuable insights into the attitudes, opinions, and emotions of the writer, and in our case, potential related underlying biases in the generated text, we conducted sentiment analysis to calculate polarity and subjectivity scores for each generated text. Polarity is a float value within the range [–1.0, 1.0], where –1.0 indicates a negative sentiment, and 1.0 a positive sentiment. Values around 0 represent a neutral sentiment. Subjectivity is a float within the range [0.0, 1.0] where 0.0 is very objective and 1.0 is very subjective. Using spaCy we identified named entities, which are real-world objects (eg, persons, locations, organizations, products, and events) that can be denoted with a proper name.

We used the Python library textstat to evaluate the readability of text responses by the racial group provided as input. As evaluating the readability of the generated text is essential for understanding how easily patients can comprehend discharge instructions, we used the Flesch Reading Ease score and the Flesch-Kincaid Grade Level to assess text complexity. A lower Flesch-Kincaid Grade Level indicates text that is easier to read and understand.

To explore if the models used certain words more frequently than others based on race/ethnicity, we calculated the word count in the output texts of each race/ethnicity. For preprocessing, we used the CountVectorizer class from the sklearn.feature_extraction.text module. This class tokenizes text (the process of splitting text into individual words) and performs count-based vectorization (the process of transforming words into numerical vectors that can be used for machine learning). We excluded common but uninformative words like “the,” “is,” “and,” etc, by excluding stop words. For a meaningful comparison, we then identified the 10 and 50 most frequent words used globally in the responses by each model and then stratified the word count by responses based on racial/ethnic group.

### Statistical Analysis

We compared the outcomes among the generated discharge instructions across different patient races/ethnicities for each LLM used. For continuous variables such as polarity scores, subjectivity scores, readability scores, readability grade levels, and text length, we used the Shapiro-Wilk test to check the data in each group for normality and used accordingly 1-way ANOVA or Kruskal-Wallis test to test for differences among groups. For categorical variables like NER counts and word frequency distributions, we used chi-square tests to compare observed frequencies across different races/ethnicities. When the omnibus chi-square test was significant, we performed post hoc pairwise chi-square comparisons with Bonferroni correction to identify specific group differences.

The chi-square test of independence was conducted to assess the relationship between different categorical variables, using the chi2_contingency function from the SciPy library. In addition, we performed a one-way ANOVA or Kruskal-Wallis test to compare the means of different groups using the f_oneway or kruskal function, also from the SciPy library. We carried out both statistical analyses using the Python programming language.

### Ethical Considerations

The study was conducted using a fully deidentified dataset that included no personal or protected health identifiers as defined under 45 CFR §46.102(f) of the US Department of Health and Human Services regulations [10]. Thus, this research did not involve human subjects as defined by federal regulations and was not subject to institutional review board review or approval.

## Results

**Figure 1:**
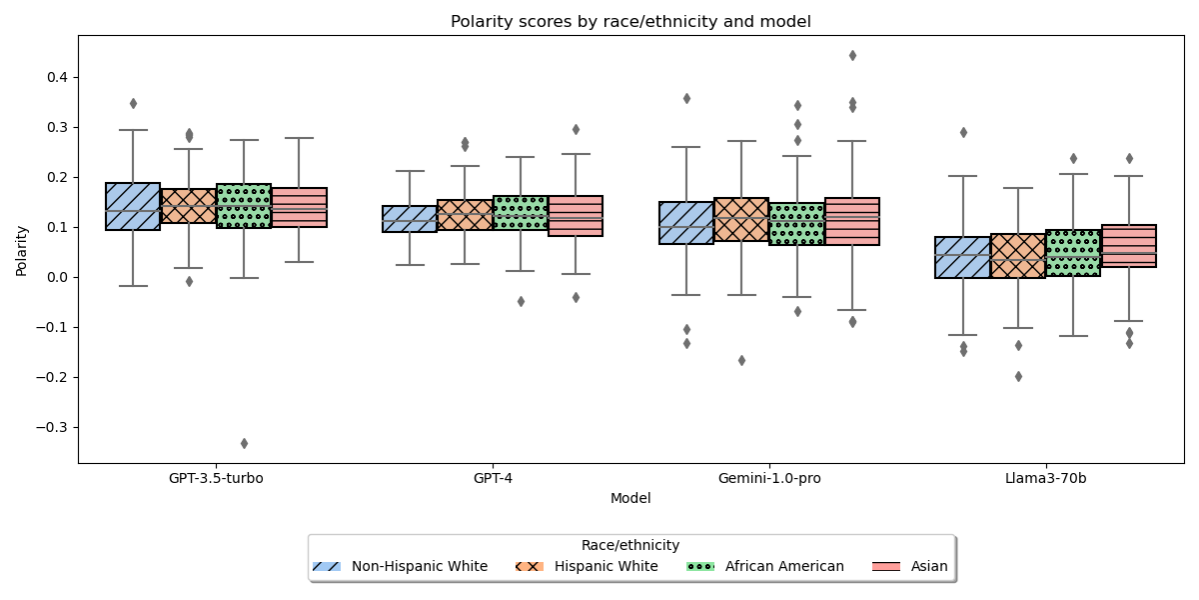
Polarity of LLM-generated text by race/ethnicity. LLM: large language model.

**Figure 2:**
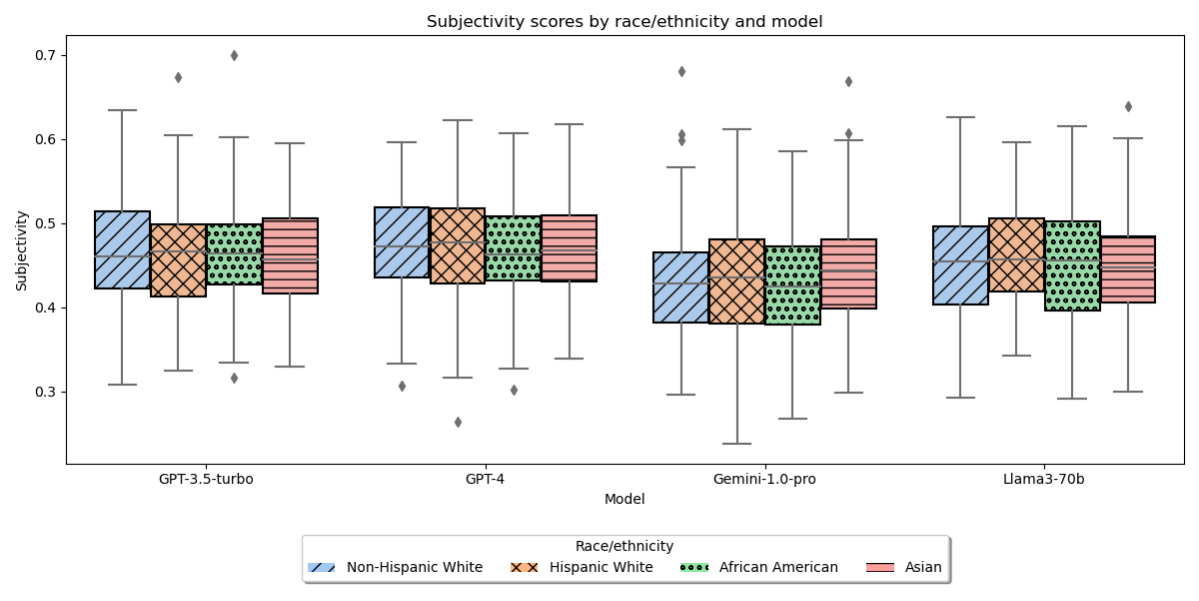
Subjectivity of LLM-generated text by race/ethnicity. LLM: large language model.

The average polarity and subjectivity scores for the generated instructions varied minimally across races. The differences in the average polarity (Figure 1) of the discharge instructions generated for African American, Asian, Hispanic White, and non-Hispanic White patients examined using Kruskal-Wallis test; H-statistics were 0.94 (*P*=.82) for GPT-3.5-turbo, 3.19 (*P*=.36) for GPT-4, 1.45 (*P*=.70) for Gemini-1.0-pro, and 3.38 (*P*=.34) for Llama3-70b. The differences in the average subjectivity (Figure 2) of the discharge instructions generated for African American, Asian, Hispanic White, and non-Hispanic White patients examined using ANOVA, F-statistics were 0.04 (*P*=.99) for GPT-3.5-turbo, 0.67 (*P*=.57) for GPT-4, 0.4 (*P*=.76) for Gemini-1.0-pro, and 0.3 (*P*=.82) for Llama3-70b.

**Figure 3:**
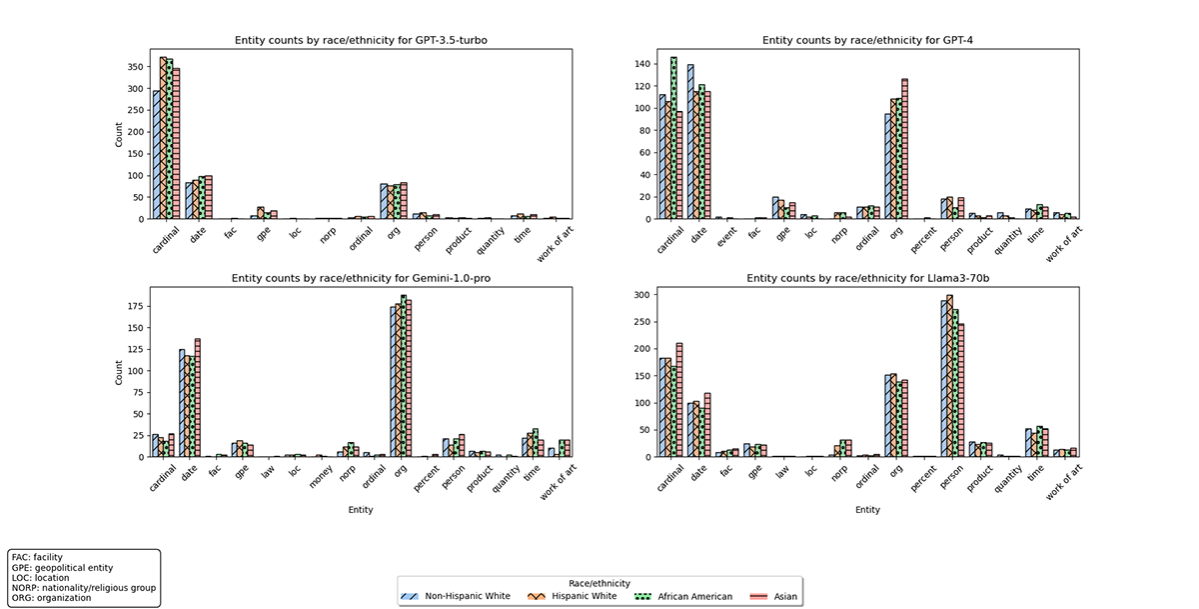
Entity counts of LLM-generated text by race/ethnicity. LLM: large language model.

We observed comparable results for the NER (Figure 3) with a *χ*^2^_36_=34.26 (*P*=.55) for GPT-3.5-turbo, *χ*^2^_42_=58.41 (*P*=.047) for GPT-4, *χ*^2^_45_=52.15 (*P*=.22) for Gemini-1.0-pro, *χ*^2^_42_=48.75 (*P*=.22) for Llama3-70b. Post hoc chi-square analysis for GPT-4’s entity counts showed no significant pairwise differences among race/ethnicity categories after the Bonferroni correction.

**Figure 4:**
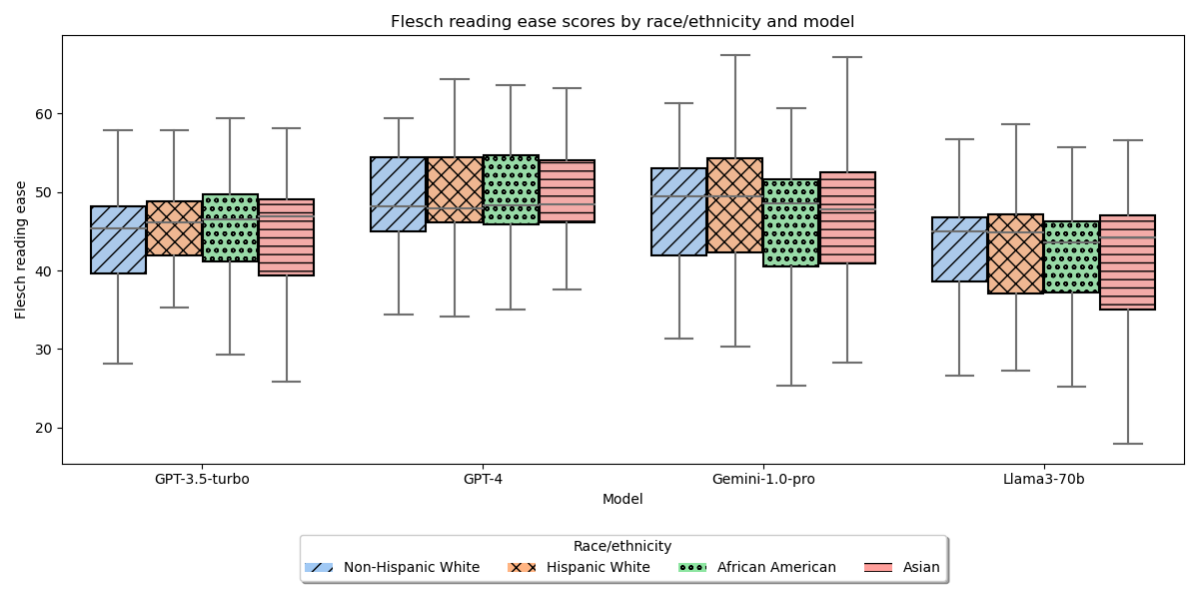
Flesch Reading ease of LLM-generated text by race/ethnicity. LLM: large language model.

**Figure 5:**
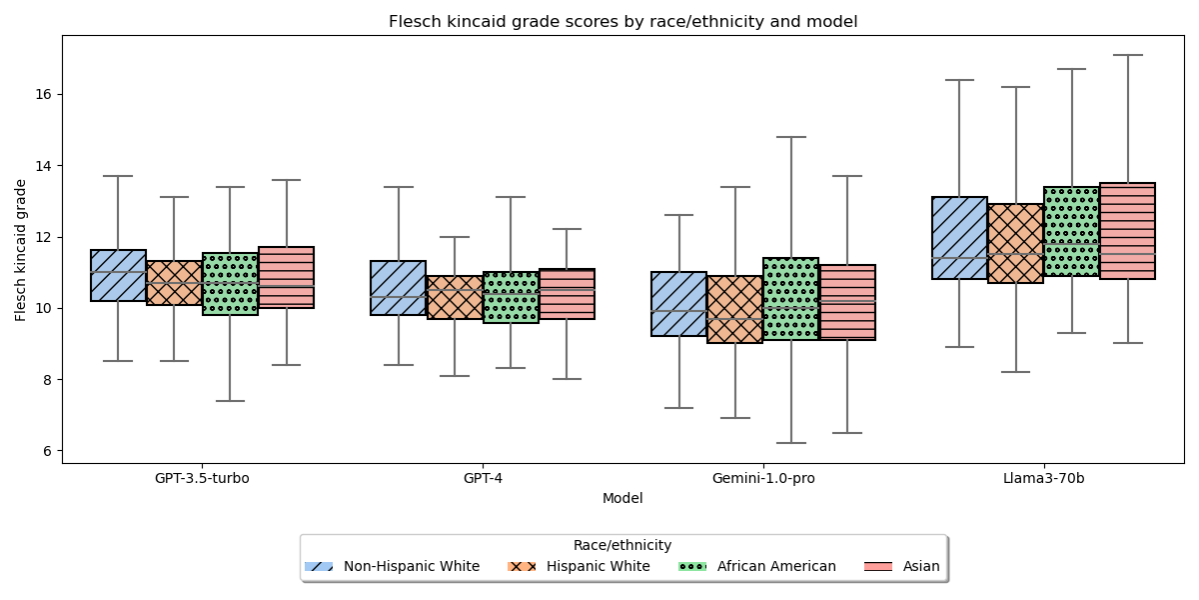
Flesch Kincaid grade of LLM-generated text by race/ethnicity. LLM: large language model.

The readability ease scores and grade levels (Figures 4 and 5) showed no significant differences across the races/ethnicities (readability scores: H=4.01, *P*=.26 for GPT=3.5-turbo; H=0.86, *P*=.83 for GPT-4; H=2.26, *P*=.52 for Gemini-1.0-pro; and H=1.59, *P*=.66 for Llama3-70b; readability grade level: H=3.41, *P*=.33 for GPT-3.5-turbo; H=1.53, *P*=.68 for GPT-4; H=2.26, *P*=.52 for Gemini-1.0-pro; and H=1.41, *P*=.7 for Llama3-70b).

**Figure 6:**
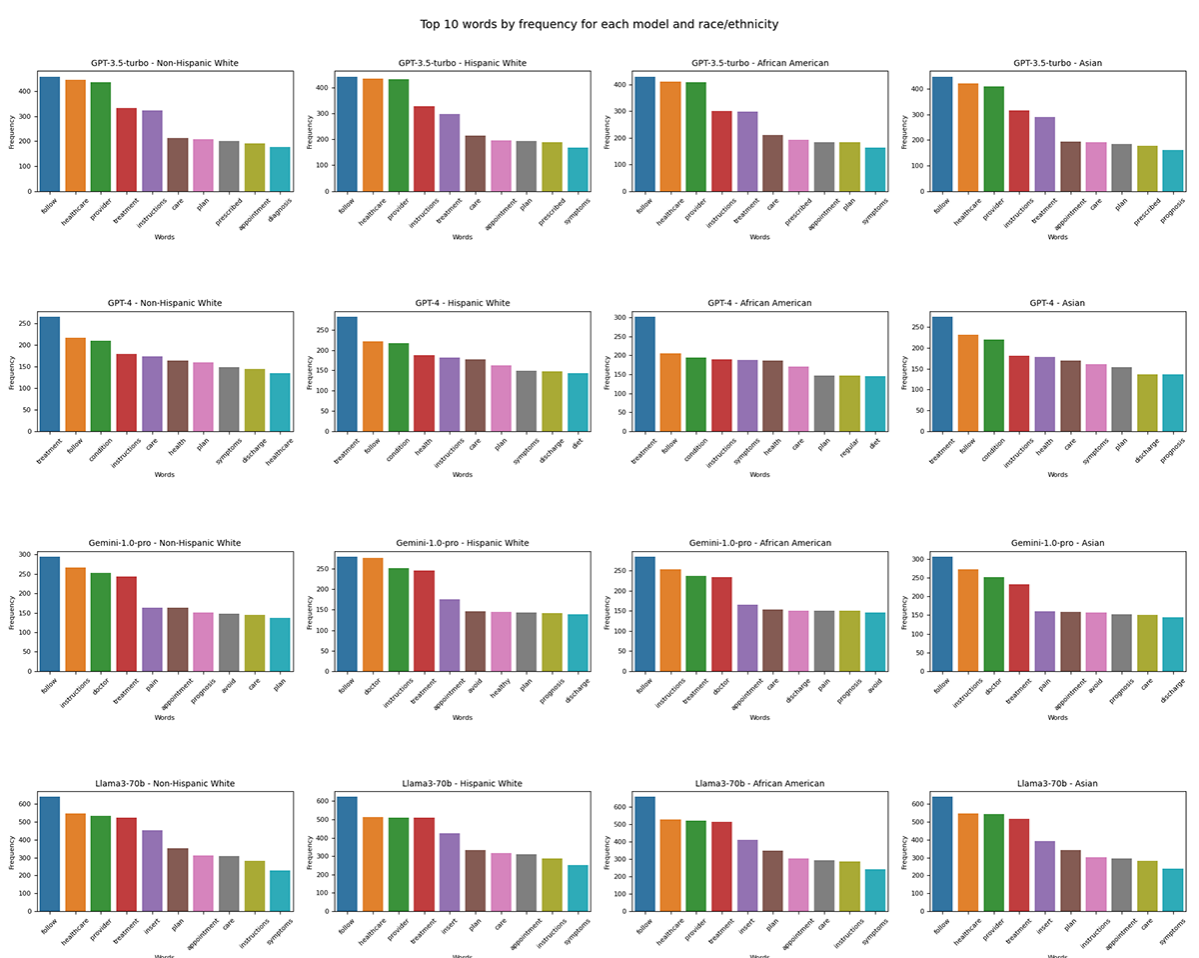
Frequency of top 10 words of LLM-generated text by race/ethnicity. LLM: large language model.

The distribution of the word frequency of the 10 most frequent words used by each model across races/ethnicities did not statistically significantly vary (*χ*^2^_27_=6.02, *P*<.99 for GPT-3.5-turbo; *χ*^2^_27_=14.87, *P*=.97 for GPT-4; *χ*^2^_27_=13.51, *P*=.99 for Gemini-1.5-pro; and *χ*^2^_27_=12.27, *P*=.99 for Llama3-70b). Similarly, no statistically significant difference was observed for the top 50 words distribution by each model across the examined races/ethnicities (*χ*^2^_147_=85.11, *P*<.99 for GPT-3.5-turbo; *χ*^2^_147_=84.09, *P*<.99 for GPT-4; *χ*^2^_147_=87.21, *P*<.99 for Gemini-1.0-pro; and *χ*^2^_147_=93.21, *P*<.99 for Llama3-70b; Figure 6).

Statistical analysis results have been included in Multimedia Appendix 2.

## Discussion

### Principal Findings

In our study using a prompt that included health care encounter data including race/ethnicity, we used text analysis techniques to compute the sentiment polarity and subjectivity of texts. We used NER to identify and categorize proper nouns and other significant terms with the corpus. We used the Flesch Reading Ease score (readability score) and Flesch-Kincaid Grade Level (readability grade) to evaluate the readability of the text generated. We also calculated the most frequently used words of generated text by four popular LLMs: GPT-3.5-turbo, GPT-4, Gemini-1.0-pro, and Llama3-70b. Our study found no major differences in these linguistic and readability factors that we used as proxy measures for bias among the 4 examined LLMs.

LLMs hold great promise in health care, facilitating various tasks with realistic and knowledge-rich responses. Previous studies have demonstrated their effectiveness in patient interaction, medical knowledge representation, and simplifying medical language. In a study of patient questions posted on social media comparing responses by physicians and a chatbot using an LLM, the bot’s responses were not only preferred over the physicians’ but also ranked higher in empathy and quality [11].

LLMs not only produce realistic text responses, but they also encode clinical and other knowledge as demonstrated by ChatGPT performing at or near passing threshold for 3 steps of the United States Medical Licensing Exam and the Clinical Informatics examination [4,12,13]. ChatGPT has also been successfully used to translate radiology reports into plain language [14]. In a study where ChatGPT was presented with advice-seeking vignettes, ChatGPT was found to “consider” social factors like race altering clinical recommendations [15].

With its use in health care–related tasks when first released to the public, the concern of racial and other biases inherent in the LLMs becomes important. When detecting a gender bias by ChatGPT, Brown et al [16] stated “Internet-trained models have internet-scale biases.” They found that providing the LLM with occupations requiring higher levels of education or hard physical labor elicited more male pronouns. Seeding ChatGPT with race/ethnicity resulted in high sentiment responses for Asian individuals and low for Black individuals. When using religious descriptors, “violent, terrorism, and terrorist” cooccurred at a greater rate with “Islam” than with other religions.

By now, most LLM developers have locked their tools against task requests that are obviously seeking to elicit bias. To circumnavigate these blocks, we analyzed health care–related text generated in simple terms by 4 LLMs where the prompts were identical except for race/ethnicity without obviously seeking to elicit bias in the prompt. The NLP linguistic factors that we used in our study as proxy measures for bias were not vastly different based on race/ethnicity. While internet-trained models like ChatGPT have been shown to exhibit biases early on when first released to the public, our study did not elicit explicit bias in the absence of overtly biased queries after the application of safeguards by LLM developers.

We used specific tools in this study to linguistically examine the models’ outputs. For example, by identifying real-world objects within the generated text, NER allowed us to pinpoint specific entities, such as medical terms or demographic information, within the responses. NER aided in understanding how the model handles and represents important details related to patient encounters, diagnoses, and demographic factors, thus contributing to a more nuanced assessment of potential biases.

In addition, we conducted a readability and sentiment analysis to understand if the models tailor their responses differently to various racial groups in terms of text complexity. Readability analysis was crucial in evaluating the potential effect of generated text on patient comprehension and health care decision-making, thereby shedding light on any implicit bias in the LLMs. Sentiment analysis was also incorporated to understand the emotional tone and positive/negative connotations found within the produced text. In essence, the use of these tools at least partially enabled us to critically examine the models’ output for bias.

Our study observed that the text generated by 4 popular LLMs exhibited no major differences across most examined surrogate linguistic metrics for racial/ethnic bias in generated responses. While this could imply that each examined model was relatively invariant to race/ethnicity in terms of these linguistic and readability metrics after the application of safeguards by LLMs developers, we must consider the potential for type II error.

### Limitations

Several limitations of this study warrant consideration. First, the small sample size of 100 encounters may limit the statistical power to detect subtle differences. Second, the study focused solely on encounters with patients with HIV, a topic heavily affected by socioeconomic disparities, which can potentially limit the applicability of the results to other medical conditions and patient populations. Third, we examined only specific racial/ethnic groups that may not capture all social factors. Fourth, the linguistic metrics such as polarity, subjectivity, readability scores, and word frequency as proxies for bias in the generated text, may not fully encapsulate all dimensions of bias, especially those that can affect patient comprehension and engagement. Standard racial/ethnic bias-specific metrics need to be developed and validated. Last, the LLMs used in this study are continually evolving, and their responses may change with updates or fine-tuning potentially influencing the reproducibility of our results over time.

The nuanced understanding of bias in artificial intelligence (AI), as evidenced by our study design and findings, underscores the critical role of technology in shaping patient interactions and treatment outcomes. By discussing instances where artificial intelligence responses may or may not vary by race, we can guide the development and deployment of more equitable artificial intelligence systems. These improvements are vital for ensuring that all patient groups receive clear, understandable, and unbiased information when using large language models, which is crucial for informed health care decision-making and equitable treatment.

### Conclusion

Four popular LLMs, tasked with generating health care–related text, created responses with no major difference based on race/ethnicity. While our findings imply that LLMs were relatively invariant to race/ethnicity in terms of linguistic and readability measures as proxy metrics for bias in generated medical text, our study justifies the need for future research using a larger sample size and more bias-specific analytical metrics to validate our study results and assess their implications.

## Data Availability

The datasets generated or analyzed during this study are available from the corresponding author on reasonable request.

## Appendix

Multimedia Appendix 1 Prompt used in the study.

Multimedia Appendix 2 Statistical analysis for each model metrics by race/ethnicity.

## Abbreviations

AI: artificial intelligence
API: application programming interface
LLM: large language model
NER: named entity recognition
NLP: natural language processing
