# Supplementary material for: Assessing Racial and Ethnic Bias in Text Generation by Large Language Models for Health Care–Related Tasks: Cross-Sectional Study
Source: J Med Internet Res. 2025 Mar 13;27:e57257. doi: 10.2196/57257 (PMC11950697; doi:10.2196/57257)
Supplement: Multimedia Appendix 2 [file jmir_v27i1e57257_app2.docx]

**Continuous Variables Descriptive Statistics:**

| LLM Model | Race/Ethnicity | Non-Hispanic White | Hispanic White | African American | Asian | P-value |
| --- | --- | --- | --- | --- | --- | --- |
| GPT-3.5-turbo | Polarity | 0.140 (0.066) | 0.146 (0.057) | 0.136 (0.072) | 0.140 (0.057) | .82 |
|  | Subjectivity | 0.462 (0.062) | 0.462 (0.061) | 0.464 (0.060) | 0.461 (0.060) | .99 |
|  | Flesch Reading Ease | 44.39 (6.40) | 46.07 (5.75) | 46.29 (7.58) | 44.99 (8.14) | .26 |
|  | Flesch Kincaid Grade | 10.98 (1.10) | 10.71 (0.98) | 10.69 (1.38) | 10.92 (1.56) | .33 |
| GPT-4 | Polarity | 0.114 (0.041) | 0.126 (0.051) | 0.125 (0.050) | 0.122 (0.054) | .36 |
|  | Subjectivity | 0.476 (0.060) | 0.474 (0.065) | 0.464 (0.060) | 0.471 (0.062) | .57 |
|  | Flesch Reading Ease | 48.61 (6.28) | 49.08 (5.78) | 49.12 (5.72) | 49.57 (5.16) | .83 |
|  | Flesch Kincaid Grade | 10.57 (1.18) | 10.40 (0.99) | 10.33 (1.05) | 10.35 (0.95) | .68 |
| Gemini-1.0-pro | Polarity | 0.103 (0.074) | 0.113 (0.067) | 47.37 (9.66) | 10.21 (2.61) | .70 |
|  | Subjectivity | 0.432 (0.073) | 0.431 (0.073) | 0.431 (0.069) | 0.440 (0.067) | .76 |
|  | Flesch Reading Ease | 47.37 (9.66) | 47.96 (9.49) | 46.61 (8.82) | 47.16 (9.78) | .52 |
|  | Flesch Kincaid Grade | 10.21 (2.61) | 10.19 (2.45) | 10.35 (2.01) | 10.24 (2.04) | .52 |
| Llama3-70b | Polarity | 0.039 (0.075) | 0.039 (0.067) | 0.044 (0.071) | 0.054 (0.071) | .34 |
|  | Subjectivity | 0.454 (0.070) | 0.459 (0.059) | 0.451 (0.074) | 0.450 (0.063) | .82 |
|  | Flesch Reading Ease | 40.47 (12.35) | 37.38 (32.49) | 39.79 (19.03) | 30.80 (50.71) | .66 |
|  | Flesch Kincaid Grade | 13.13 (4.28) | 14.34 (12.24) | 13.35 (7.13) | 16.78 (19.50) | .70 |

- Mean (SD)

**Categorical Variables Descriptive Statistics:**

**Entity Counts:**

**GPT-3.5-turbo:**

| **Entity** | **Non-Hispanic White** | **Hispanic White** | **African American** | **Asian** |
| --- | --- | --- | --- | --- |
| CARDINAL | 295 (59%) | 372 (61%) | 368 (63%) | 346 (60%) |
| DATE | 84 (17%) | 89 (15%) | 98 (17%) | 99 (17%) |
| ORG | 80 (16%) | 77 (13%) | 79 (14%) | 84 (15%) |
| PERSON | 12 (2%) | 15 (2%) | 7 (1%) | 11 (2%) |
| GPE | 8 (2%) | 27 (4%) | 14 (2%) | 19 (3%) |
| TIME | 7 (1%) | 12 (2%) | 6 (1%) | 10 (2%) |
| ORDINAL | 3 (1%) | 6 (1%) | 4 (1%) | 6 (1%) |
| PRODUCT | 3 (1%) | 1 (0%) | 3 (1%) | 1 (0%) |
| WORK_OF_ART | 2 (0%) | 4 (1%) | 2 (0%) | 2 (0%) |
| NORP | 2 (0%) | 1 (0%) | 2 (0%) | 1 (0%) |
| QUANTITY | 1 (0%) | 3 (0%) | 1 (0%) | 0 (0%) |
| LOC | 0 (0%) | 1 (0%) | 0 (0%) | 0 (0%) |
| FAC | 0 (0%) | 0 (0%) | 1 (0%) | 0 (0%) |

- Count (proportion)

**GPT-4:**

| Entity | Non-Hispanic White | Hispanic White | African American | Asian |
| --- | --- | --- | --- | --- |
| DATE | 139 (33%) | 115 (29%) | 121 (28%) | 115 (29%) |
| CARDINAL | 112 (26%) | 106 (26%) | 146 (33%) | 97 (24%) |
| ORG | 95 (22%) | 108 (27%) | 109 (25%) | 126 (31%) |
| GPE | 20 (5%) | 17 (4%) | 10 (2%) | 15 (4%) |
| PERSON | 18 (4%) | 20 (5%) | 10 (2%) | 19 (5%) |
| ORDINAL | 11 (3%) | 11 (3%) | 12 (3%) | 11 (3%) |
| TIME | 9 (2%) | 8 (2%) | 13 (3%) | 11 (3%) |
| QUANTITY | 6 (1%) | 3 (1%) | 1 (0%) | 0 (0%) |
| WORK_OF_ART | 6 (1%) | 4 (1%) | 5 (1%) | 2 (0%) |
| PRODUCT | 5 (1%) | 3 (1%) | 1 (0%) | 3 (1%) |
| LOC | 4 (1%) | 2 (0%) | 3 (1%) | 0 (0%) |
| EVENT | 2 (0%) | 0 (0%) | 1 (0%) | 0 (0%) |
| NORP | 0 (0%) | 6 (1%) | 6 (1%) | 2 (0%) |
| FAC | 0 (0%) | 0 (0%) | 1 (0%) | 1 (0%) |
| PERCENT | 0 (0%) | 0 (0%) | 1 (0%) | 0 (0%) |

- Count (proportion)

**Gemini-1.0-pro:**

| Entity | Non-Hispanic White | Hispanic White | African American | Asian |
| --- | --- | --- | --- | --- |
| ORG | 174 (42%) | 178 (44%) | 188 (42%) | 182 (40%) |
| DATE | 125 (30%) | 118 (29%) | 117 (26%) | 137 (30%) |
| CARDINAL | 26 (6%) | 23 (6%) | 18 (4%) | 27 (6%) |
| TIME | 22 (5%) | 28 (7%) | 33 (7%) | 20 (4%) |
| PERSON | 21 (5%) | 14 (0%) | 25 (5%) | 26 (6%) |
| GPE | 16 (4%) | 19 (5%) | 16 (4%) | 14 (3%) |
| WORK_OF_ART | 10 (2%) | 3 (1%) | 20 (4%) | 20 (4%) |
| PRODUCT | 7 (2%) | 5 (1%) | 7 (2%) | 6 (1%) |
| NORP | 6 (1%) | 12 (3%) | 17 (4%) | 12 (3%) |
| ORDINAL | 5 (1%) | 1 (0%) | 2 (0%) | 3 (1%) |
| LOC | 2 (0%) | 2 (0%) | 3 (1%) | 2 (0%) |
| QUANTITY | 2 (0%) | 0 (0%) | 2 (0%) | 1 (0%) |
| FAC | 1 (0%) | 0 (0%) | 3 (1%) | 2 (0%) |
| PERCENT | 0 (0%) | 1 (0%) | 0 (0%) | 3 (1%) |
| MONEY | 0 (0%) | 2 (0%) | 1 (0%) | 0 (0%) |
| LAW | 0 (0%) | 0 (0%) | 0 (0%) | 1 (0%) |

- Count (proportion)

**Llama3-70b:**

| Entity | Non-Hispanic White | Hispanic White | African American | Asian |
| --- | --- | --- | --- | --- |
| PERSON | 289 (34%) | 299 (34%) | 273 (32%) | 246 (28%) |
| CARDINAL | 182 (21%) | 182 (21%) | 168 (20%) | 210 (24%) |
| ORG | 151 (18%) | 154 (17%) | 139 (16%) | 142 (16%) |
| DATE | 100 (12%) | 103 (12%) | 90 (11%) | 118 (13%) |
| TIME | 52 (6%) | 44 (5%) | 57 (7%) | 52 (6%) |
| PRODUCT | 28 (3%) | 23 (3%) | 27 (3%) | 26 (3%) |
| GPE | 24 (3%) | 19 (2%) | 23 (3%) | 22 (2%) |
| WORK_OF_ART | 13 (2%) | 14 (2%) | 13 (2%) | 17 (2%) |
| FAC | 8 (1%) | 11 (1%) | 13 (2%) | 15 (2%) |
| QUANTITY | 4 (0%) | 2 (0%) | 2 (0%) | 1 (0%) |
| NORP | 4 (0%) | 21 (2%) | 32 (4%) | 31 (3%) |
| ORDINAL | 3 (0%) | 4 (0%) | 3 (0%) | 5 (1%) |
| PERCENT | 2 (0%) | 1 (0%) | 1 (0%) | 2 (0%) |
| LAW | 1 (0%) | 1 (0%) | 1 (0%) | 1 (0%) |
| LOC | 0 (0%) | 2 (0%) | 1 (0%) | 1 (0%) |

- Count (proportion)

Distribution of entity counts by each model across race/ethnicity categories:

| Model | Chi-Square Statistic | Degree of Freedom | P-value |
| --- | --- | --- | --- |
| GPT-3.5-turbo | 34.26 | 36 | .55 |
| GPT-4 | 58.41 | 42 | .047 |
| Gemini-1.0-pro | 52.15 | 45 | .22 |
| Llama3-70b | 48.75 | 42 | .22 |

Entity counts post hoc analysis:
Pairwise Chi-Square post hoc results for GPT-4:

| Pair | Chi-Square Statistic | p (original) | p (Bonferroni-corrected) | Reject H0H_0H0​ |
| --- | --- | --- | --- | --- |
| Hispanic White vs. African American | 16.60 | .28 | 1.00 | No |
| Hispanic White vs. Asian | 11.07 | .52 | 1.00 | No |
| Hispanic White vs. Non-Hispanic White | 13.56 | .33 | 1.00 | No |
| African American vs. Asian | 23.89 | .05 | .28 | No |
| African American vs. Non-Hispanic White | 27.69 | .02 | .09 | No |
| Asian vs. Non-Hispanic White | 25.40 | .02 | .12 | No |

**Word Frequency:**

Count and proportion of the top 50 words used by GPT-3.5-turbo stratified by race/ethnicity

| Word | Non-Hispanic White | Hispanic White | African American | Asian |
| --- | --- | --- | --- | --- |
| follow | 458 (0.028) | 442 (0.027) | 429 (0.027) | 448 (0.028) |
| healthcare | 445 (0.027) | 436 (0.026) | 412 (0.026) | 423 (0.026) |
| provider | 437 (0.027) | 432 (0.026) | 408 (0.025) | 411 (0.026) |
| instructions | 324 (0.020) | 329 (0.020) | 301 (0.019) | 317 (0.020) |
| treatment | 333 (0.020) | 299 (0.018) | 298 (0.019) | 290 (0.018) |
| care | 212 (0.013) | 214 (0.013) | 210 (0.013) | 191 (0.012) |
| plan | 207 (0.013) | 193 (0.012) | 183 (0.011) | 185 (0.012) |
| appointment | 192 (0.012) | 195 (0.012) | 184 (0.011) | 193 (0.012) |
| prescribed | 201 (0.012) | 189 (0.011) | 192 (0.012) | 177 (0.011) |
| prognosis | 174 (0.011) | 165 (0.010) | 151 (0.009) | 162 (0.010) |
| symptoms | 166 (0.010) | 169 (0.010) | 164 (0.010) | 147 (0.009) |
| diagnosis | 177 (0.011) | 134 (0.008) | 147 (0.009) | 161 (0.010) |
| important | 153 (0.009) | 161 (0.010) | 150 (0.009) | 153 (0.010) |
| avoid | 155 (0.010) | 162 (0.010) | 155 (0.010) | 145 (0.009) |
| medications | 154 (0.009) | 157 (0.009) | 155 (0.010) | 147 (0.009) |
| discharge | 132 (0.008) | 133 (0.008) | 122 (0.008) | 125 (0.008) |
| pain | 114 (0.007) | 144 (0.009) | 123 (0.008) | 117 (0.007) |
| self | 124 (0.008) | 127 (0.008) | 116 (0.007) | 123 (0.008) |
| condition | 102 (0.006) | 123 (0.007) | 135 (0.008) | 115 (0.007) |
| concerns | 111 (0.007) | 115 (0.007) | 114 (0.007) | 111 (0.007) |
| diet | 116 (0.007) | 105 (0.006) | 118 (0.007) | 111 (0.007) |
| general | 105 (0.006) | 106 (0.006) | 102 (0.006) | 107 (0.007) |
| healthy | 99 (0.006) | 97 (0.006) | 111 (0.007) | 111 (0.007) |
| specific | 102 (0.006) | 103 (0.006) | 97 (0.006) | 113 (0.007) |
| admitted | 101 (0.006) | 102 (0.006) | 101 (0.006) | 102 (0.006) |
| personalized | 107 (0.007) | 106 (0.006) | 91 (0.006) | 91 (0.006) |
| monitor | 105 (0.006) | 96 (0.006) | 91 (0.006) | 99 (0.006) |
| recommended | 92 (0.006) | 90 (0.005) | 95 (0.006) | 97 (0.006) |
| based | 115 (0.007) | 83 (0.005) | 82 (0.005) | 91 (0.006) |
| maintain | 87 (0.005) | 88 (0.005) | 90 (0.006) | 89 (0.006) |
| urgency | 87 (0.005) | 87 (0.005) | 81 (0.005) | 91 (0.006) |
| schedule | 86 (0.005) | 81 (0.005) | 92 (0.006) | 84 (0.005) |
| infection | 87 (0.005) | 87 (0.005) | 77 (0.005) | 74 (0.005) |
| regular | 80 (0.005) | 78 (0.005) | 68 (0.004) | 95 (0.006) |
| help | 75 (0.005) | 87 (0.005) | 74 (0.005) | 80 (0.005) |
| questions | 77 (0.005) | 79 (0.005) | 81 (0.005) | 79 (0.005) |
| directed | 81 (0.005) | 72 (0.004) | 86 (0.005) | 73 (0.005) |
| health | 76 (0.005) | 73 (0.004) | 89 (0.006) | 70 (0.004) |
| good | 77 (0.005) | 85 (0.005) | 70 (0.004) | 73 (0.005) |
| medication | 70 (0.004) | 72 (0.004) | 96 (0.006) | 67 (0.004) |
| manage | 74 (0.005) | 66 (0.004) | 75 (0.005) | 85 (0.005) |
| rest | 65 (0.004) | 76 (0.005) | 69 (0.004) | 74 (0.005) |
| experience | 75 (0.005) | 71 (0.004) | 65 (0.004) | 66 (0.004) |
| support | 70 (0.004) | 74 (0.004) | 69 (0.004) | 62 (0.004) |
| proper | 66 (0.004) | 73 (0.004) | 74 (0.005) | 58 (0.004) |
| stay | 57 (0.004) | 71 (0.004) | 74 (0.005) | 67 (0.004) |
| ensure | 64 (0.004) | 63 (0.004) | 73 (0.005) | 68 (0.004) |
| prevent | 65 (0.004) | 70 (0.004) | 67 (0.004) | 63 (0.004) |
| smoking | 65 (0.004) | 54 (0.003) | 66 (0.004) | 77 (0.005) |
| balanced | 69 (0.004) | 64 (0.004) | 59 (0.004) | 68 (0.004) |

Count and proportion of the top 50 words used by GPT-4 stratified by race/ethnicity

| Word | Non-Hispanic White | Hispanic White | African American | Asian |
| --- | --- | --- | --- | --- |
| treatment | 265 (0.016) | 282 (0.017) | 302 (0.018) | 275 (0.017) |
| follow | 217 (0.013) | 222 (0.014) | 206 (0.012) | 232 (0.014) |
| condition | 209 (0.013) | 217 (0.013) | 195 (0.012) | 220 (0.013) |
| instructions | 179 (0.011) | 181 (0.011) | 189 (0.011) | 182 (0.011) |
| health | 163 (0.010) | 187 (0.011) | 187 (0.011) | 179 (0.011) |
| care | 174 (0.011) | 177 (0.011) | 171 (0.010) | 170 (0.010) |
| symptoms | 148 (0.009) | 149 (0.009) | 188 (0.011) | 161 (0.010) |
| plan | 159 (0.010) | 163 (0.010) | 147 (0.009) | 154 (0.009) |
| discharge | 144 (0.009) | 147 (0.009) | 146 (0.009) | 137 (0.008) |
| diet | 130 (0.008) | 143 (0.009) | 146 (0.009) | 135 (0.008) |
| healthcare | 134 (0.008) | 139 (0.008) | 135 (0.008) | 124 (0.008) |
| prognosis | 128 (0.008) | 136 (0.008) | 131 (0.008) | 137 (0.008) |
| regular | 127 (0.008) | 127 (0.008) | 147 (0.009) | 124 (0.008) |
| help | 121 (0.007) | 112 (0.007) | 141 (0.008) | 130 (0.008) |
| appointment | 116 (0.007) | 127 (0.008) | 117 (0.007) | 124 (0.008) |
| prescribed | 113 (0.007) | 132 (0.008) | 122 (0.007) | 113 (0.007) |
| smoking | 118 (0.007) | 122 (0.007) | 124 (0.007) | 115 (0.007) |
| pain | 128 (0.008) | 118 (0.007) | 110 (0.007) | 120 (0.007) |
| medications | 122 (0.007) | 119 (0.007) | 110 (0.007) | 118 (0.007) |
| healthy | 118 (0.007) | 110 (0.007) | 116 (0.007) | 116 (0.007) |
| provider | 110 (0.007) | 118 (0.007) | 113 (0.007) | 115 (0.007) |
| avoid | 114 (0.007) | 112 (0.007) | 111 (0.007) | 111 (0.007) |
| maintain | 107 (0.006) | 108 (0.007) | 103 (0.006) | 114 (0.007) |
| self | 110 (0.007) | 107 (0.007) | 105 (0.006) | 101 (0.006) |
| therapy | 97 (0.006) | 116 (0.007) | 93 (0.006) | 108 (0.007) |
| admitted | 101 (0.006) | 103 (0.006) | 100 (0.006) | 101 (0.006) |
| continue | 94 (0.006) | 109 (0.007) | 95 (0.006) | 98 (0.006) |
| general | 96 (0.006) | 95 (0.006) | 103 (0.006) | 101 (0.006) |
| monitor | 90 (0.005) | 91 (0.006) | 101 (0.006) | 99 (0.006) |
| art | 91 (0.006) | 99 (0.006) | 87 (0.005) | 102 (0.006) |
| doctor | 102 (0.006) | 94 (0.006) | 103 (0.006) | 76 (0.005) |
| important | 92 (0.006) | 82 (0.005) | 94 (0.006) | 103 (0.006) |
| medication | 85 (0.005) | 89 (0.005) | 102 (0.006) | 76 (0.005) |
| remember | 83 (0.005) | 89 (0.005) | 93 (0.006) | 78 (0.005) |
| diagnosis | 76 (0.005) | 82 (0.005) | 101 (0.006) | 69 (0.004) |
| weight | 79 (0.005) | 78 (0.005) | 76 (0.005) | 86 (0.005) |
| balanced | 73 (0.004) | 83 (0.005) | 76 (0.005) | 78 (0.005) |
| medical | 77 (0.005) | 69 (0.004) | 81 (0.005) | 68 (0.004) |
| manage | 61 (0.004) | 65 (0.004) | 84 (0.005) | 81 (0.005) |
| exercise | 69 (0.004) | 67 (0.004) | 78 (0.005) | 70 (0.004) |
| overall | 64 (0.004) | 76 (0.005) | 59 (0.004) | 74 (0.005) |
| recovery | 75 (0.005) | 69 (0.004) | 64 (0.004) | 59 (0.004) |
| infection | 71 (0.004) | 66 (0.004) | 78 (0.005) | 50 (0.003) |
| lifestyle | 60 (0.004) | 66 (0.004) | 71 (0.004) | 68 (0.004) |
| make | 61 (0.004) | 64 (0.004) | 71 (0.004) | 65 (0.004) |
| body | 71 (0.004) | 61 (0.004) | 73 (0.004) | 55 (0.003) |
| antiretroviral | 59 (0.004) | 69 (0.004) | 64 (0.004) | 64 (0.004) |
| physical | 62 (0.004) | 63 (0.004) | 61 (0.004) | 68 (0.004) |
| heart | 54 (0.003) | 60 (0.004) | 75 (0.004) | 65 (0.004) |
| ensure | 61 (0.004) | 57 (0.003) | 65 (0.004) | 62 (0.004) |

Count and proportion of the top 50 words used by Gemini-1.0-pro stratified by race/ethnicity

| Word | Non-Hispanic White | Hispanic White | African American | Asian |
| --- | --- | --- | --- | --- |
| follow | 294 (0.023) | 279 (0.022) | 284 (0.022) | 306 (0.024) |
| instructions | 266 (0.020) | 252 (0.020) | 253 (0.020) | 272 (0.021) |
| doctor | 253 (0.019) | 276 (0.022) | 234 (0.018) | 252 (0.019) |
| treatment | 243 (0.019) | 245 (0.019) | 237 (0.019) | 232 (0.018) |
| appointment | 163 (0.012) | 175 (0.014) | 165 (0.013) | 159 (0.012) |
| pain | 164 (0.013) | 136 (0.011) | 150 (0.012) | 161 (0.012) |
| avoid | 148 (0.011) | 146 (0.011) | 146 (0.011) | 157 (0.012) |
| prognosis | 151 (0.012) | 142 (0.011) | 150 (0.012) | 152 (0.012) |
| care | 145 (0.011) | 134 (0.011) | 153 (0.012) | 151 (0.012) |
| discharge | 128 (0.010) | 139 (0.011) | 150 (0.012) | 144 (0.011) |
| plan | 137 (0.010) | 143 (0.011) | 132 (0.010) | 135 (0.010) |
| healthy | 132 (0.010) | 144 (0.011) | 136 (0.011) | 132 (0.010) |
| symptoms | 125 (0.010) | 110 (0.009) | 125 (0.010) | 132 (0.010) |
| medications | 103 (0.008) | 136 (0.011) | 116 (0.009) | 129 (0.010) |
| diet | 108 (0.008) | 117 (0.009) | 120 (0.009) | 117 (0.009) |
| self | 106 (0.008) | 104 (0.008) | 109 (0.009) | 105 (0.008) |
| plenty | 92 (0.007) | 103 (0.008) | 109 (0.009) | 95 (0.007) |
| prescribed | 104 (0.008) | 103 (0.008) | 100 (0.008) | 92 (0.007) |
| smoking | 100 (0.008) | 96 (0.008) | 98 (0.008) | 97 (0.007) |
| exercise | 99 (0.008) | 94 (0.007) | 96 (0.007) | 95 (0.007) |
| diagnosis | 87 (0.007) | 86 (0.007) | 80 (0.006) | 86 (0.007) |
| general | 91 (0.007) | 81 (0.006) | 82 (0.006) | 80 (0.006) |
| eat | 75 (0.006) | 82 (0.006) | 85 (0.007) | 86 (0.007) |
| rest | 69 (0.005) | 77 (0.006) | 84 (0.007) | 80 (0.006) |
| weeks | 78 (0.006) | 80 (0.006) | 74 (0.006) | 78 (0.006) |
| alcohol | 83 (0.006) | 78 (0.006) | 73 (0.006) | 72 (0.006) |
| manage | 81 (0.006) | 73 (0.006) | 73 (0.006) | 79 (0.006) |
| help | 75 (0.006) | 80 (0.006) | 73 (0.006) | 74 (0.006) |
| experience | 79 (0.006) | 70 (0.006) | 73 (0.006) | 79 (0.006) |
| need | 81 (0.006) | 76 (0.006) | 67 (0.005) | 71 (0.005) |
| infection | 68 (0.005) | 75 (0.006) | 80 (0.006) | 67 (0.005) |
| people | 72 (0.006) | 78 (0.006) | 68 (0.005) | 70 (0.005) |
| monitor | 76 (0.006) | 72 (0.006) | 68 (0.005) | 70 (0.005) |
| improve | 71 (0.005) | 65 (0.005) | 64 (0.005) | 79 (0.006) |
| stress | 72 (0.006) | 58 (0.005) | 64 (0.005) | 76 (0.006) |
| admission | 65 (0.005) | 61 (0.005) | 65 (0.005) | 78 (0.006) |
| contact | 70 (0.005) | 68 (0.005) | 63 (0.005) | 62 (0.005) |
| important | 62 (0.005) | 59 (0.005) | 57 (0.004) | 66 (0.005) |
| regularly | 58 (0.004) | 53 (0.004) | 75 (0.006) | 56 (0.004) |
| healthcare | 66 (0.005) | 49 (0.004) | 53 (0.004) | 64 (0.005) |
| regular | 56 (0.004) | 65 (0.005) | 51 (0.004) | 60 (0.005) |
| health | 62 (0.005) | 54 (0.004) | 56 (0.004) | 53 (0.004) |
| condition | 53 (0.004) | 55 (0.004) | 58 (0.005) | 55 (0.004) |
| provider | 59 (0.005) | 48 (0.004) | 50 (0.004) | 62 (0.005) |
| directed | 58 (0.004) | 55 (0.004) | 54 (0.004) | 51 (0.004) |
| fluids | 49 (0.004) | 56 (0.004) | 58 (0.005) | 53 (0.004) |
| proper | 52 (0.004) | 54 (0.004) | 55 (0.004) | 54 (0.004) |
| medical | 45 (0.003) | 53 (0.004) | 65 (0.005) | 51 (0.004) |
| patient | 43 (0.003) | 39 (0.003) | 58 (0.005) | 74 (0.006) |
| medication | 64 (0.005) | 36 (0.003) | 59 (0.005) | 50 (0.004) |

Count and proportion of the top 50 words used by Llama3-70b stratified by race/ethnicity

| Word | Non-Hispanic White | Hispanic White | African American | Asian |
| --- | --- | --- | --- | --- |
| follow | 639 (0.032) | 623 (0.032) | 656 (0.033) | 640 (0.033) |
| healthcare | 546 (0.028) | 514 (0.027) | 528 (0.027) | 549 (0.028) |
| provider | 533 (0.027) | 509 (0.026) | 520 (0.026) | 545 (0.028) |
| treatment | 524 (0.027) | 509 (0.026) | 514 (0.026) | 518 (0.027) |
| insert | 451 (0.023) | 424 (0.022) | 410 (0.021) | 392 (0.020) |
| plan | 353 (0.018) | 335 (0.017) | 347 (0.017) | 343 (0.018) |
| appointment | 311 (0.016) | 311 (0.016) | 302 (0.015) | 296 (0.015) |
| care | 310 (0.016) | 316 (0.016) | 293 (0.015) | 281 (0.014) |
| instructions | 283 (0.014) | 288 (0.015) | 286 (0.014) | 301 (0.015) |
| symptoms | 229 (0.012) | 251 (0.013) | 241 (0.012) | 238 (0.012) |
| contact | 214 (0.011) | 205 (0.011) | 221 (0.011) | 215 (0.011) |
| patient | 223 (0.011) | 199 (0.010) | 202 (0.010) | 203 (0.010) |
| concerns | 219 (0.011) | 191 (0.010) | 193 (0.010) | 210 (0.011) |
| monitor | 188 (0.010) | 186 (0.010) | 191 (0.010) | 207 (0.011) |
| condition | 198 (0.010) | 196 (0.010) | 181 (0.009) | 190 (0.010) |
| scheduled | 200 (0.010) | 175 (0.009) | 182 (0.009) | 206 (0.011) |
| date | 216 (0.011) | 202 (0.010) | 179 (0.009) | 159 (0.008) |
| discharge | 196 (0.010) | 189 (0.010) | 184 (0.009) | 181 (0.009) |
| ensure | 186 (0.009) | 176 (0.009) | 189 (0.010) | 176 (0.009) |
| avoid | 163 (0.008) | 177 (0.009) | 191 (0.010) | 185 (0.009) |
| information | 185 (0.009) | 173 (0.009) | 187 (0.009) | 163 (0.008) |
| medications | 176 (0.009) | 172 (0.009) | 174 (0.009) | 181 (0.009) |
| manage | 160 (0.008) | 161 (0.008) | 176 (0.009) | 151 (0.008) |
| attend | 168 (0.009) | 154 (0.008) | 161 (0.008) | 152 (0.008) |
| self | 155 (0.008) | 148 (0.008) | 160 (0.008) | 159 (0.008) |
| questions | 157 (0.008) | 135 (0.007) | 152 (0.008) | 144 (0.007) |
| prognosis | 140 (0.007) | 136 (0.007) | 153 (0.008) | 154 (0.008) |
| hesitate | 148 (0.007) | 131 (0.007) | 139 (0.007) | 135 (0.007) |
| directed | 146 (0.007) | 133 (0.007) | 126 (0.006) | 140 (0.007) |
| appointments | 125 (0.006) | 119 (0.006) | 130 (0.007) | 136 (0.007) |
| birth | 148 (0.007) | 135 (0.007) | 114 (0.006) | 102 (0.005) |
| prescribed | 117 (0.006) | 120 (0.006) | 119 (0.006) | 115 (0.006) |
| diagnosis | 107 (0.005) | 110 (0.006) | 128 (0.006) | 105 (0.005) |
| time | 121 (0.006) | 105 (0.005) | 99 (0.005) | 113 (0.006) |
| pain | 105 (0.005) | 101 (0.005) | 116 (0.006) | 113 (0.006) |
| general | 98 (0.005) | 108 (0.006) | 117 (0.006) | 109 (0.006) |
| changes | 105 (0.005) | 108 (0.006) | 96 (0.005) | 101 (0.005) |
| proper | 91 (0.005) | 95 (0.005) | 86 (0.004) | 92 (0.005) |
| medication | 97 (0.005) | 77 (0.004) | 86 (0.004) | 96 (0.005) |
| hospital | 103 (0.005) | 81 (0.004) | 83 (0.004) | 85 (0.004) |
| recovery | 84 (0.004) | 90 (0.005) | 78 (0.004) | 95 (0.005) |
| stay | 93 (0.005) | 90 (0.005) | 80 (0.004) | 78 (0.004) |
| reach | 102 (0.005) | 74 (0.004) | 85 (0.004) | 78 (0.004) |

Word Frequency analysis for top 10 words by each model across races/ethnicities

| **Model** | **Top 10 Words** | **Chi-Square Statistic** | **Degrees of Freedom** | **p-value** |
| --- | --- | --- | --- | --- |
| GPT-3.5-turbo | follow, healthcare, provider, instructions, treatment, care, plan, appointment, prescribed, prognosis | 6.02 | 27 | 1.00 |
| GPT-4 | treatment, follow, condition, instructions, health, care, symptoms, plan, discharge, diet | 14.87 | 27 | .97 |
| Gemini-1.0-pro | follow, instructions, doctor, treatment, appointment, pain, avoid, prognosis, care, discharge | 13.51 | 27 | .99 |
| Llama3-70b | follow, healthcare, provider, treatment, insert, plan, appointment, care, instructions, symptoms | 12.27 | 27 | .99 |

Word Frequency analysis for top 50 words by each model across races/ethnicities

| **Model** | **Top 50 Words** | **Chi-Square Statistic** | **Degrees of Freedom** | **p-value** |
| --- | --- | --- | --- | --- |
| **GPT-3.5-turbo** | follow, healthcare, provider, instructions, treatment, care, plan, appointment, prescribed, prognosis, symptoms, diagnosis, avoid, important, medications, discharge, pain, self, condition, concerns, diet, general, healthy, specific, admitted, personalized, monitor, recommended, based, maintain, urgency, schedule, infection, regular, help, questions, directed, health, good, medication, manage, rest, experience, support, proper, stay, ensure, prevent, smoking, balanced | 85.11 | 147 | 1.00 |
| **GPT-4** | treatment, follow, condition, instructions, health, care, symptoms, plan, discharge, diet, healthcare, prognosis, regular, help, appointment, prescribed, smoking, pain, medications, healthy, provider, avoid, maintain, self, therapy, admitted, continue, general, monitor, art, doctor, important, medication, remember, diagnosis, weight, balanced, medical, manage, exercise, overall, recovery, infection, lifestyle, make, body, antiretroviral, heart, physical, ensure | 84.09 | 147 | 1.00 |
| **Gemini-1.0-pro** | follow, instructions, doctor, treatment, appointment, pain, avoid, prognosis, care, discharge, plan, healthy, symptoms, medications, diet, self, plenty, prescribed, smoking, exercise, diagnosis, general, eat, rest, weeks, alcohol, manage, help, experience, need, infection, people, monitor, improve, stress, admission, contact, important, regularly, healthcare, regular, health, condition, provider, directed, fluids, proper, medical, patient, medication | 87.21 | 147 | 1.00 |
| **Llama3-70b** | follow, healthcare, provider, treatment, insert, plan, appointment, care, instructions, symptoms, contact, patient, concerns, monitor, condition, scheduled, date, discharge, ensure, avoid, information, medications, manage, attend, self, questions, prognosis, hesitate, directed, appointments, birth, prescribed, diagnosis, time, pain, general, changes, progress, additional, complications, prevent, experience, remember, diet, proper, medication, hospital, recovery, stay, reach | 93.21 | 147 | 1.00 |
